# Transformation of MCF-10A cells by random mutagenesis with frameshift mutagen ICR191: A model for identifying candidate breast-tumor suppressors

**DOI:** 10.1186/1476-4598-7-51

**Published:** 2008-06-05

**Authors:** Helena Zientek-Targosz, Dimiter Kunnev, Lesleyann Hawthorn, Mikhail Venkov, Sei-Ichi Matsui, Richard T Cheney, Yuri Ionov

**Affiliations:** 1 Department of Cancer Genetics, Roswell Park Cancer Institute, Buffalo, New York 14263, USA; 2 Department of Pathology and Laboratory Medicine, Roswell Park Cancer Institute, Buffalo, New York 14263, USA

## Abstract

**Background:**

Widely accepted somatic mutation theory of carcinogenesis states that mutations in oncogenes and tumor suppressor genes in genomes of somatic cells is the cause of neoplastic transformation. Identifying frequent mutations in cancer cells suggests the involvement of mutant genes in carcinogenesis.

**Results:**

To develop an in vitro model for the analysis of genetic alterations associated with breast carcinogenesis, we used random mutagenesis and selection of human non-tumorigenic immortalized breast epithelial cells MCF-10A in tissue-culture conditions that mimic tumor environment. Random mutations were generated in MCF-10A cells by cultivating them in a tissue-culture medium containing the frameshift-inducing agent ICR191. The first selective condition we used to transform MCF1-10A cells was cultivation in a medium containing mutagen at a concentration that allowed cell replication despite p53 protein accumulation induced by mutagen treatment. The second step of selection was either cell cultivation in a medium with reduced growth-factor supply or in a medium that mimics a hypoxia condition or growing in soft agar. Using mutagenesis and selection, we have generated several independently derived cultures with various degrees of transformation. Gene Identification by Nonsense-mediated mRNA decay Inhibition (GINI) analysis has identified the ICR191-induced frameshift mutations in the TP53, smoothelin, Ras association (RalGDS/AF-6) domain family 6 (RASSF6) and other genes in the transformed MCF-10A cells. The TP53 gene mutations resulting in the loss of protein expression had been found in all independently transformed MCF-10A cultures, which form large progressively growing tumors with sustained angiogenesis in nude mice.

**Conclusion:**

Identifying genes containing bi-allelic ICR191-induced frameshift mutations in the transformed MCF-10A cells generated by random mutagenesis and selection indicates putative breast-tumor suppressors. This can provide a model for studying the role of mutant genes in breast carcinogenesis.

## Background

It has been proposed that, even from its earliest stages, cancer development is associated with DNA replication stress, which leads to genomic instability and selective pressure for p53 inactivation [1]. This hypothesis was based on the fact of frequent inactivation of the p53 pathway in human tumors and on a demonstration that human early pre-cancerous lesions show signs of DNA damage-response activation [1-4]. These observations suggest that selection for cells that can tolerate DNA damage-signaling in precancerous cells is likely to take place during the early stages of tumor progression *in vivo*. We hypothesized that an extended *in vitro *cultivation of human immortalized cells in tissue culture conditions that generate DNA damage response activation and create selective pressure for p53 inactivation can result in tumorigenic cell transformation.

MCF-10A cells are frequently used as a normal control in breast cancer studies [5-9]. These cells were derived from the mammary tissue of a cystic fibrosis patient and have normal mammary epithelial cell morphology. These cells do not have mutations in the p53 gene but show homozygous loss of the p16/p15 locus[10] and do not form tumors in nude mice or colonies in semi-solid low melting agarose. Similar to normal human breast epithelial cells, at confluence the MCF-10A cells form dome structures in tissue culture plates and produce mammary spheres in 3D collagen culture. All of these characteristics make MCF-10A cells a model of choice for breast tumor progression studies.

The role of genomic instability in carcinogenesis is thought to generate variability within the tumor cell population and to facilitate selection of genetic variants that have growth advantages in the tumor environment. We speculated that continuous chronic exposure of cells to mutagens at concentrations that induce activation of the DNA damage-response, although still allowing DNA replication and cell proliferation, would provide conditions for both, mimicking genomic instability through the increased rate of mutations and selecting genetic variants that acquire tolerance to DNA damage-response activation. To mimic genomic instability and generate genetic variability within populations of MCF-10A cells, we used the acridine mutagen ICR191. This DNA-intercalating chemical produces an insertion of one G:C base-pair in a short poly-G:C repetitive DNA sequence[11]. We rationalized that frameshift mutations produced by ICR191 treatment may cause the inactivation of tumor-suppressor genes related to breast carcinogenesis and result in the transformation of MCF-10A cells. Frameshift mutations, which generate premature translation-termination codons (TPC) located more than 25 base pairs upstream of the last exon/exon junction, frequently initiate mutant-mRNA degradation through the nonsense-mediated mRNA decay (NMD) mechanism[12,13]. Thus, the mutant gene can be identified in the transformed cells using GINI analysis[14], as we have shown in prostate and colon cancer cell lines [15,16]. Bi-allelic inactivation of a gene in the transformed MCF10-A cultures may indicate a potential tumor-suppressor gene related to breast carcinogenesis.

## Results

### Transformation of MCF-10A cells using ICR191 treatment

**Figure 1:**
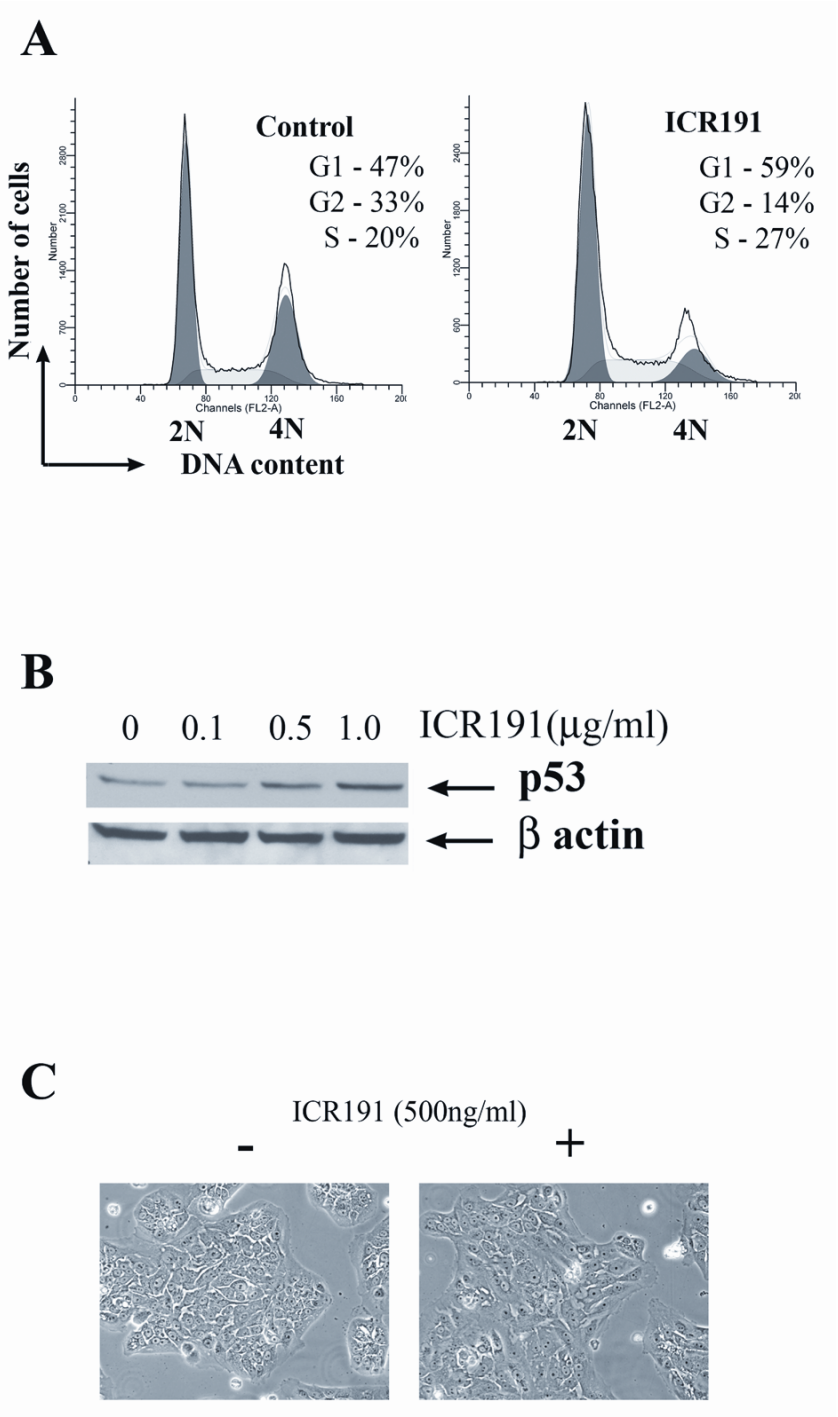
**Effect of ICR191 exposure on MCF-10A cells**. **A. **Fluorescence-activated cell-sorting (FACS) analysis demonstrates that MCF-10A cells, after 24 hours of cultivation in a tissue-culture medium containing 500 ng/ml of ICR191, have an increased proportion of cells arrested in the S and G1 phases of the cell cycle. **B**. Western blot demonstrates the accumulation of P53 protein induced by ICR191 treatment. **C**. A slightly larger size and flatter appearance of MCF-10A cells after 24 hours of exposure to 500 ng/ml of ICR191.

Mutagenesis and selection was initiated in five parallel tissue-culture plates seeded with 1 million MCF-10A cells in each plate (subsequently referred to as MCF10Aα, MCF10Aβ, MCF10Aγ, MCF10Aδ and MCF10Aε). As a result, five independent sets of MCF-10A cultures with various degrees of transformation were generated. Two consecutive steps of *in vitro *selection were used to transform the MCF-10A cells. The first selective step promoted clonal expansion of cells that had acquired tolerance to DNA damage-signaling which was achieved by cell cultivation in a complete medium containing 500 ng/ml of ICR191. This concentration of drugs allowed cell proliferation despite the DNA damage-response activation manifested as an increased level of TP53 protein, alterations in cell-cycle progression and changes in cell morphology in tissue culture, which was manifested as a slightly larger cell size and more flat appearance (Figure 1A–C).

**Figure 2:**
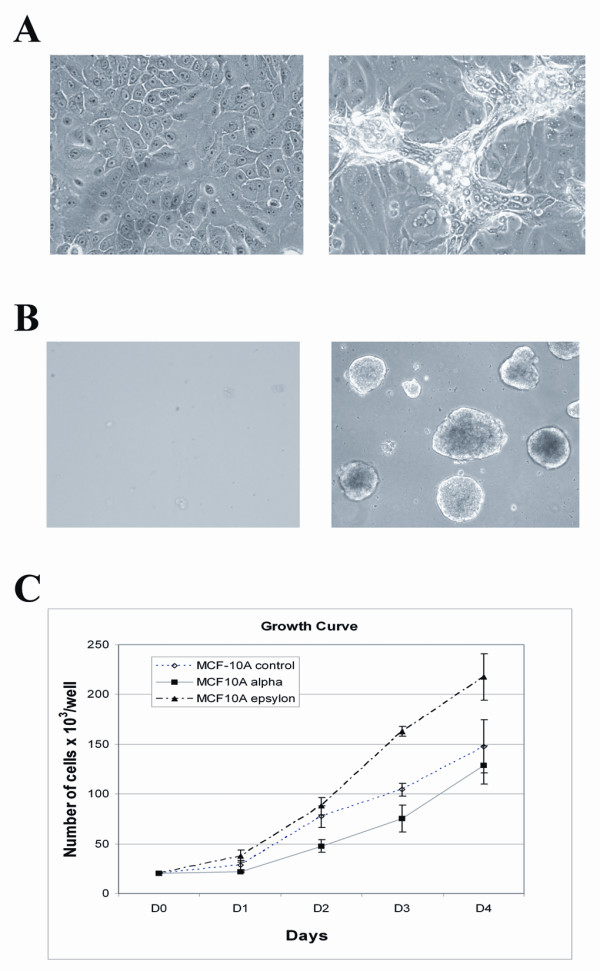
**Transformation of cells selected for the ability to replicate under chronic exposure to ICR191**. **A**. Loss of contact inhibition by the MCF-10A cells selected for DNA damage-resistance. Passage- matched control MCF-10A cells (left) and the cells from the MCF-10A alpha culture after six months of cultivation in the presence of ICR191 (right) were plated in the medium without carcinogens and allowed to grow until the state of confluence. The 3D structures formed by DNA damage-resistant at the state of confluence cells are seen under a light microscope. **B**. Microscopically visualized colonies produced in three weeks in soft agar by the MCF10Aα cells selected for resistance to ICR191 (right) but not by the control passage-matched MCF-10A cells (left). **C**. A higher rate of MCF10Aε cell-replication after cultivation for four months in the presence of ICR191. Passage-matched control MCF-10A cells and the cells from the MCF10Aα and MCF10Aε cultures after four months of cultivation in the presence of ICR191 were seeded in 30 mm plates in triplicate (5 × 10^4 ^cells per plate), and the cell numbers were counted at the indicated times using a hemocytometer. Standard errors were calculated on the basis of triplicate measurements.

After six months of selection for the resistance to DNA damage-signaling, all five cultures had a higher than control untreated cells rate of replication in the presence of mutagen (not shown). Also, all five cultures had acquired signs of cell transformation, such as the loss of contact inhibition at high density and the ability to form growing colonies in soft agar (Figures 2A and 2B). One culture (MCF10Aε) has acquired a higher rate of cell proliferation in the absence of mutagen (Figure 2C). Following subcutaneous injections of 5 million cells into the flanks of nude mice, three out of five cultures (MCF10Aα, MCF10Aγ and MCF10Aδ) produced small (~2 mm in diameter) palpable tumors. These tumors were localized at the site of inoculation for more than four months without an increase in size.

The second step of selection used to isolate more aggressive variants of the MCF-10A cells involved selection for either the ability of anchorage-independent growth or the ability to grow in a medium with either reduced growth-factor supply or resistance to hypoxia. Selection for anchorage-independent growth was applied to the MCF10Aα and MCF10Aβ cultures. Cells from these cultures had been cloned in soft agar and, after two weeks, four of the fastest-growing colonies of MCF10Aα cells, and one colony of MCF10Aβ cells, were isolated and expanded in culture. After subcutaneous injections of 5 million of these cells into the flanks of nude mice, the soft agar-derived clone of MCF10Aβ cells (MCF10Aβ1) produced a large (~1 cm in diameter), progressively growing tumor four months after inoculation. Two soft agar-derived clones from the MCF10α culture, MCF10α3 and MCF10α4, produced large and small tumors, respectively.

**Figure 3:**
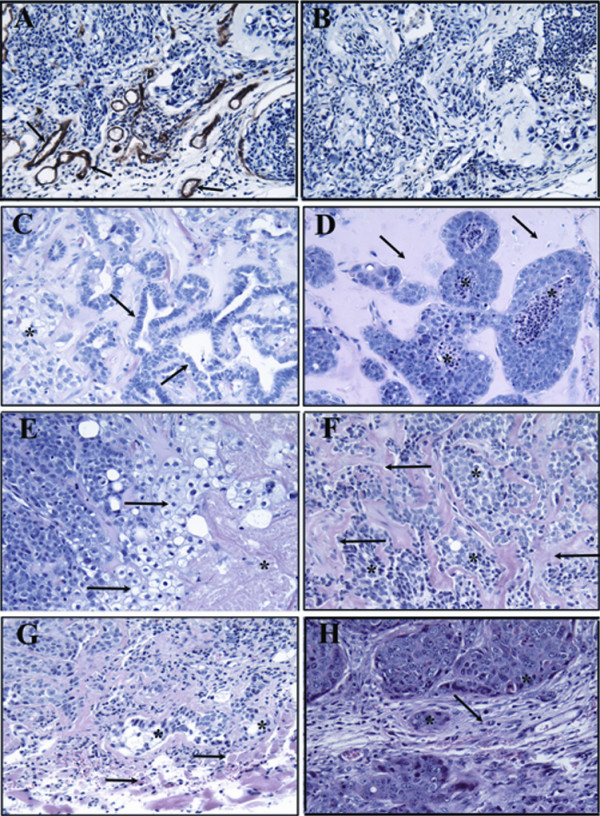
**Histological analyses of tumors produced by transformed MCF10A cells in nude mice**. The first row from the top shows neovascularity in the peritumoral stroma of a large tumor formed by transformed MCF10A cells in nude mice.**A**. Positive immunoreactivity with anti-CD31 antibody (DAB chromagen) from tumors formed in nude mice by MCF10AβL cells selected for hypoxia resistance. **B**. Negative staining with a control IgG antibody. The second and third rows from the top show variable degrees of tumor differentiation by transformed MCF10A cells. **C**. H&E section of MCF10AαL tumor cells, which were selected for reduced growth factor supply shows focal clear cell change (*) with adjacent prominent ductal differentiation (see arrows). **D**. H&E section of a tumor formed in a nude mouse by MCF10AαL cells selected for hypoxia resistance exhibit growth within desmoplastic stroma with central comedo-like tumor necrosis. **E**. H&E section of a tumor formed by the secondary inoculation of MCF10AαL cells selected for reduced growth factor supply shows an area of geographic necrosis (*) with adjacent clear cell change (arrow) and nested growth of pleomorphic cells. **F**. H&E section of another tumor formed by secondary inoculation of MCF10AαL cells selected for reduced growth factor supply shows nested and strand-like growth of cells (*) within a desmoplastic stroma (arrows). The last row from the top shows the invasiveness of tumors formed by transformed MCF10A cells. **G**. H&E section of a tumor formed by MCF10AαL shows that the tumor (*) begins to infiltrate into the adjacent skeletal muscle (arrow). **H**. H&E section shows that the pleomorphic carcinoma formed by the secondary injection of MCF10AβL cells selected for resistance to hypoxia grows within angiogenic stroma with vascular dilation and inflammation. All tissue was formalin-fixed and paraffin embedded. All images are at 20× original magnification.

After selecting for the ability to grow in the medium with reduced growth-factor supply, two of the five cultures (MCF10Aα and MCF10Aβ) acquired the ability to form large, progressively growing tumors, and three cultures (MCF10Aγ, MCF10Aδ and MCF10Aε) produced small tumors four months after inoculation. After selecting for resistance to hypoxia, three cultures (MCF10α, MCF10Aβ and MCF10Aγ) produced large tumors, and the remaining two cultures (MCF10δ and MCF10ε) produced small tumors. Large tumors produced by transformed cultures were shown to have undergone extreme angiogenesis (Figure 3A) and showed different degrees of differentiation and invasiveness (Figure 3C–H).

Individual cell-lines were established from both large and small tumors and expanded in culture. The names of cell-lines derived from large and small tumors are subsequently denoted with the letters L and S, respectively. Inoculations of cell-lines derived from large tumors resulted in the formation of large tumors (1 cm in diameter) in five out of five mice after one month. In contrast, inoculations of cell-lines derived from the small tumors produced either small tumors or none at all.

### Spectral Karyotyping (SKY) and array comparative genomic hybridization of transformed cells shows minimal chromosomal instability

**Figure 4:**
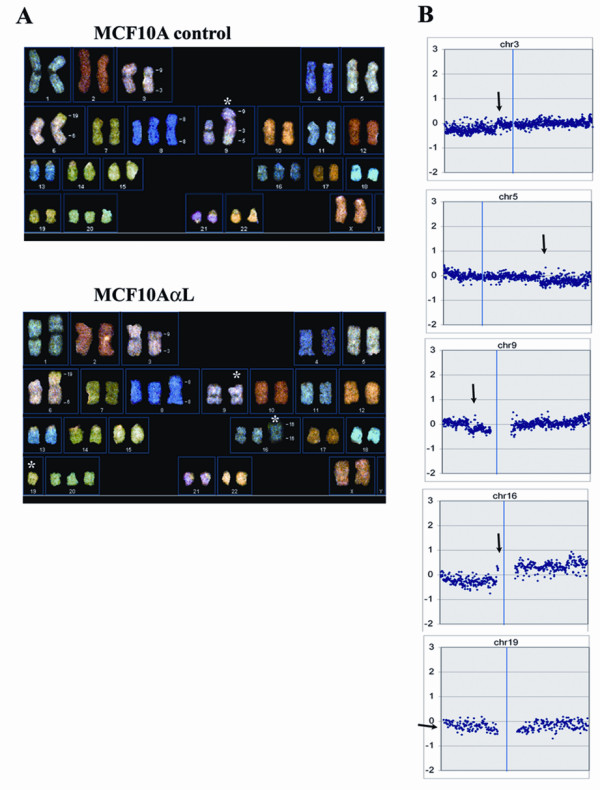
**Spectral karyotyping and array comparative genomic hybridization analyses of transformed MCF-10A cells derived from large tumors grown in nude mice. A**. SKY analysis of control MCF-10A and transformed MCF10AαL cells. *sign shows chromosomal alterations between control and transformed cells. **B**. Array CGH of MCf10AαL cells hybridized against control MCF-10A cells shows chromosomal alterations (indicated by arrows) correlated with those identified by SKY.

To identify chromosomal changes associated with the transformation of MCF-10A cells, we compared the karyotype of the transformed MCF10AαL culture derived from the tumor grown in nude mice with that of control MCF-10A cells using SKY. Control MCF-10A cells are almost diploid cells with several chromosomal abnormalities. All MCF-10A cells from our lab have a reciprocal t(3;9)(p14;p21) translocation and an unbalanced translocation from chromosome 5q to the derivative 9[10]. Loss of the p16 locus associated with t(3;9) translocation is believed to contribute to the immortalization of MCF-10A cells[17]. Also, a t(6;19)(p25;q12) rearrangement can be seen in all control MCF-10A cells used in our lab (Figure 4A). In addition, trisomies of chromosomes 20 and 16, as well as an additional structurally abnormal derivative of chromosome 8, were present in all control MCF-10A cells. Few additional chromosomal alterations have occurred in the transformed cells. Figure 4A shows the duplication of a 16q arm and the loss of one copy of a 16p arm in MCF10AαL cells. The other alteration that has occurred in transformed MCF10AαL cells was the loss of one copy of chromosome 19. Also, one derivative, which includes the translocation of chromosomes 9, 3 and 5, has been lost in the transformed MCF10AαL. A comparison of MCF10AαL cells versus control MCF-10A cells using array comparative genomic hybridization (aCGH) has also identified the duplication of 16q, the loss of part of 16p and the loss of t(9;3;5) derivative in transformed cells (Figure 4B).

### GINI analysis identifies ICR191-induced mutations in the transformed MCF-10A cells

**Figure 5:**
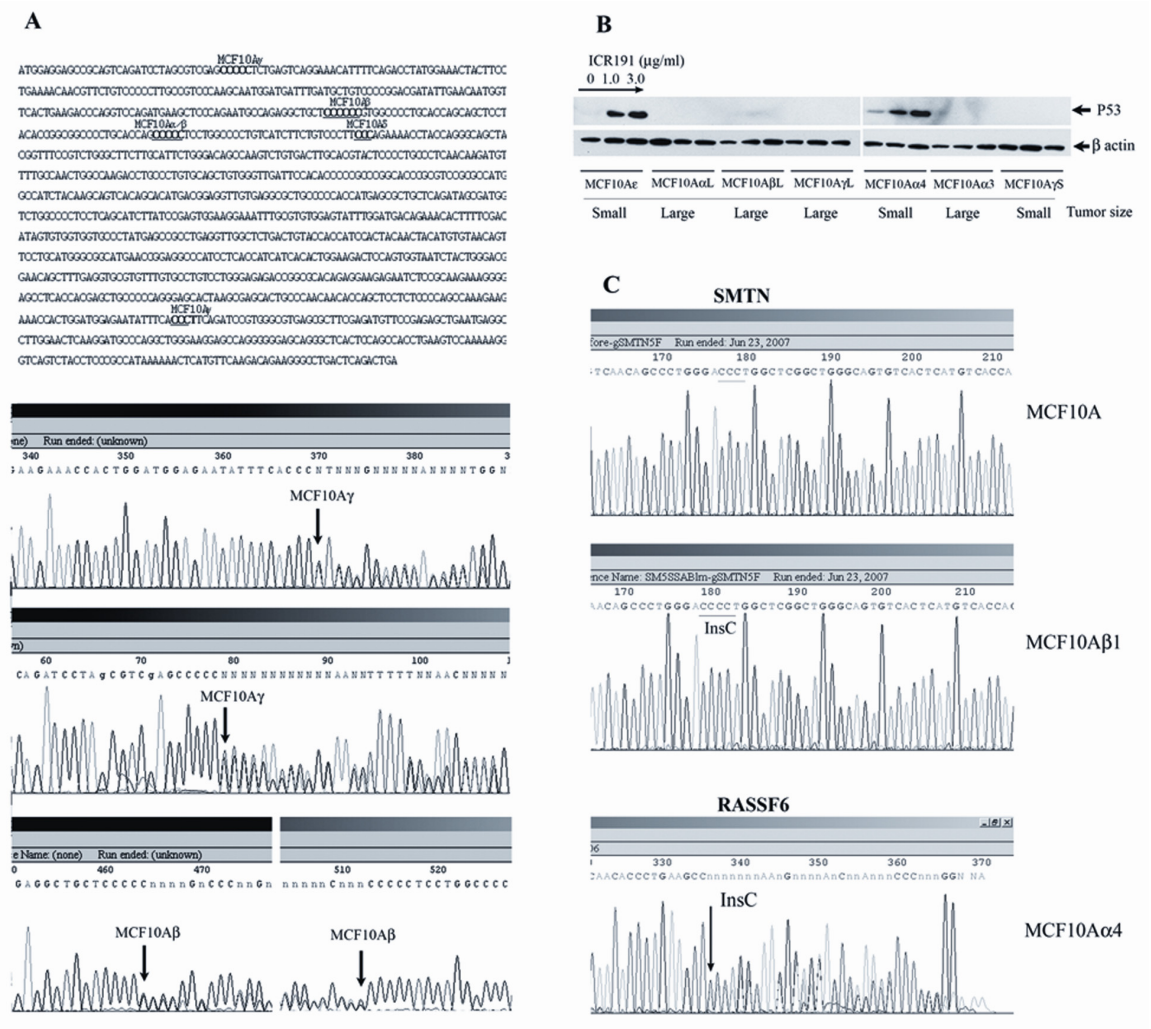
**ICR191-induced frameshift mutations identified using inhibition of NMD and microarray analysis in the p53 and other genes in the transformed MCF-10A cells. A**. Top: P53 gene-coding sequence with the frameshift mutations sites in different MCF-10A cultures underlined. Bottom: Sequence analysis shows heterozygous insertions of one cytosine within different short poly-C repeats in the coding DNA of p53 gene in MCF10Aγ and MCF10Aβ cells. In the MCF10Aβ cells, both mutations can be seen in the sequencing graph of a single PCR amplified fragment. The insertion of one C in the poly-C repeat located at 460–464 nucleotides of the fragment results in the appearance of double peaks on the sequencing graph. The second insertion in the poly-C repeat at nucleotides 512–517 of the second allele results in the disappearance of double peaks at the end of the sequencing graph. **B**. Western blotting analysis of p53 protein expression and accumulation in response to ICR191 exposure in different MCF-10A derivative cell-lines. Only those cell-lines with the loss of p53 expression could form large, progressively growing tumors in nude mice. **C**. Top: Sequencing graphs show the homozygous insertion of one C into the coding poly-C of the SMTN gene. (C)_4 _(mutant) and (C)_3 _(wild type) repeats can be seen on the sequences of SMTN gene from the MCF-10A and MCF10Aβ1 cells, respectively. Bottom: Sequencing graph shows the heterozygous insertion of one C into the coding poly-C repeat, which results in the appearance of double peaks at the end of the sequenced fragment of a RASSF6 gene in the MCF10Aα4 cells.

To identify mutations that might have contributed to MCF-10A cell-transformation, we used GINI analysis first on the MCF10Aβ1 cells, as described in Material and Methods. These cells, which were isolated from the colony growing in soft agar, produce large, continuously growing tumors in nude mice, indicating that breast cancer-related tumor-suppressors could be mutated and selected during transformation. Analyzing levels of mRNA transcripts in the MCF10Aβ1 and control MCF1-10A cells using Affymetrix U133Plus2.0 GeneChips before and after inhibition of NMD with emetine, we have selected 14 genes that satisfied the requirements for candidates for sequencing analysis (see Material and Methods). The candidates included the genes coding for TP53, smoothelin (SMTN) and ubiquitin specific peptidase 10 (USP10), which contained insertions of one cytosine in short coding poly-cytosine repeats, which are the characteristic mutations induced by ICR191. The TP53 gene in the MCF10Aβ1 cells contained two heterozygous mutations, resulting in the absence of p53 protein expression (Figure 5A), while the SMTN (Figure 5C) and UPS10 (not shown) genes had homozygous and heterozygous mutations, respectively.

The TP53 gene is known to be frequently mutated in more than half of all human cancers, including breast cancer. Since the TP53 gene is the main regulator of DNA damage-response[18] and the transformed derivatives of MCF-10A cells had been selected for the ability to replicate despite DNA damage-response activation, we analyzed all the transformed MCF-10A cultures for the mutations in the p53 gene. We found that all MCF-10A cultures that can produce large, continuously growing tumors in nude mice contained p53 gene mutations. The MCF10AγL culture had two heterozygous mutations that were different from those in the MCF10β1 cells (Figure 5A), and the MCF10AαL culture had one heterozygous mutation that was identical to one mutation found in the MCF10 β1 cells. Although the mutation had been identified only in one allele, the MCF10AαL cells, similarly to the MCF10AγL and MCF10β1, do not express p53 protein (Figure 5B). The DNA sequencing analysis of the p53 gene in MCF10Aα3 and MCF10Aα4 cells generated from soft agar growing clones has identified the mutation identical to that in MCF10AαL cells. Western blot analysis has shown that MCF10Aα3 cells, which can produce large tumors after inoculations into nude mice, do not express p53 protein, while the MCF10Aα4, which cannot produce large tumors, do. Similarly, MCF10Aδ and MCF10Aε cultures, which, after all rounds of selection, could form only small tumors in nude mice, had a wild-type allele of the p53 gene and showed p53 protein-accumulation in response to treatment with ICR191 (Figure 5B). On the other hand, the MCF10AγS cells, which do not express p53 protein due to mutations identical to that in MCF10AγL cells, did not produce large tumors in nude mice, suggesting that p53 inactivation was necessary but not sufficient for *in vivo *tumor growth.

In contrast to the p53 gene mutations, the mutation in the SMTN gene had been found only in the MCF10Aβ1 cells. All other transformed MCF-10A cultures, including the related MCF10AβL cell-line derived from a large tumor produced by MCF10Aβ cells selected for resistance to hypoxia, contained wild-type alleles of this gene. To identify other genes mutated in the process of *in vitro *transformation, we analyzed, using GINI, the MCF10Aα4 and MCF10αL cultures. Sequencing of selected candidate genes has identified heterozygous frameshift mutations in the Ras association (RalGDS/AF-6) domain family 6 (RASSF6) (Figure 5C) in the MCF10Aα4 cells and in the kinesin family member 22 (KIF22) in the MCF10αL cells.

## Discussion

It has been hypothesized that the selection of genetic or epigenetic DNA alterations, which provide growth/survival advantages to somatic cells in the context of the tumor environment, is the driving force for tumor progression. We have demonstrated that the simulation of Darwinian evolution *in vitro *by generating random mutations and selecting in tissue-culture conditions that mimic the tumor environment can result in the tumorigenic transformation of non-tumorigenic cells. Due to the stochastic nature of mutations, all the transformed cultures of the MCF-10A cells had various degrees of tumorigenicity. Only three out of five cultures, which had been subjected to random mutagenesis and selection, have acquired the ability to form large, continuously growing tumors with sustained angiogenesis in nude mice, despite the fact that all five cultures were treated identically. All three cultures had gained inactivating mutations in the TP53 gene, resulting in the loss of protein expression. TP53 gene mutations identified in the transformed MCF10AαL, MCF10AβL and MCF10AγL cells derived from tumors growing in nude mice were identical to the mutations identified in the corresponding cultures derived after *in vitro *selection either for resistance to hypoxia or to reduced growth-factor supply, or after selection for growth in soft agar. This result suggests that in each transformed culture TP53-inactivating mutations had occurred during the first round of selection when cells were cultivated in the presence of ICR191, and that the progenitors of rare cells that had acquired TP53 gene mutations underwent clonal expansion during the second round of selection in tissue-culture conditions that simulated tumor environment.

ICR191-induced frameshift mutations generate premature translation termination codons (PTC), which frequently initiate mutant mRNA degradations *via *a nonsense-mediated mRNA decay (NMD) pathway. Genes mutated in the cells transformed by ICR191 exposure can be identified using microarray-based analysis of mRNA levels alterations induced by the inhibition of NMD in the transformed as well as in the non-transformed control cells. Identifying genes in the transformed cells containing either inactivating mutations in both alleles or a mutation in one allele accompanied by the loss of the second allele can indicate potential tumor suppressors. Bi-allelic mutations of the TP53 gene identified in the transformed MCF10Aβ1 cells using NMD inhibition demonstrate the proof-of-principle that our model of cell-transformation by ICR191 exposure can be used for the identification of breast tumor suppressors.

The RASS6 gene was the other known tumor-suppressor gene that has been identified using NMD-inhibition to contain frameshift mutation in the clone of transformed MCF-10A cells derived from a colony growing in soft agar. The expression of this gene has been shown to be down-regulated in 30–60% of tumor-derived tissues from the breast, colon, kidney, liver, rectum, pancreas, stomach and thyroid gland compared to normal tissue [19]. Due to mono-allelic occurrence in the transformed MCF-10A cells, the mutation in the RASS6 gene, as well as the heterozygous mutations in USP10 and KIF22 genes, can be passenger mutations not relevant to cell transformation. We cannot rule out the possibility, however, that haploinsufficiency caused by RASSF6 heterozygous mutation could contribute to the survival of the MCF10Aα4 clone in soft agar.

Smoothelin was the gene identified by NMD-inhibition to contain homozygous ICR191-induced mutation in the MCF10Aβ1 cells. The protein encoded by this gene associates with stress fibers and constitutes part of the cytoskeleton. Since the SMTN gene mutation has been identified in a clone of MCF10Aβ cells selected for the ability to grow in soft agar, inactivation of this gene could promote anchorage-independent growth, a property of tumor cells allowing them to grow in improper locations *in vivo*, a characteristic that distinguishes malignant from benign tumors[20]. Interestingly, SMTN gene mutations have recently been identified in colorectal tumors, and the gene has been suggested as a candidate colorectal cancer gene[21]. We are planning to use MCF10Aβ1 cells, which are null for the SMTN gene expression, as a model to study the possible role of the gene in breast carcinogenesis.

## Conclusion

We have demonstrated that by using NMD-inhibition and microarray-based gene expression analysis we can identify mutant genes in tumorigenic cells transformed by random mutagenesis and selection. This strategy can be applied to the identification of genes and pathways controlling particular traits of cell transformation. For example, similar to identifying TPP53 gene mutations after selecting for tolerance to DNA damage-response activation, GINI analysis of clones isolated after random mutagenesis and selection of cells for resistance to chemotherapeutic drugs can result in the identification of other genes controlling response to chemotherapy. Also, our model of random mutagenesis and selection can be used for microarray-based monitoring of gene-expression alterations accompanying progression from almost normal to a transformed phenotype of breast epithelial cells.

## Methods

### Cell-culture media and chemicals

The immortalized, non-transformed human mammary epithelial cell-line, MCF-10A, was grown on standard tissue-culture plastic in a 5% CO_2_-humidified incubator at 37°C in Complete MCF-10A Growth Medium composed of DMEM/F12 (Mediatech, Inc., Herndon, VA) supplemented with 5% donor horse serum, 20 ng/ml epidermal growth factor (EGF), 10 μg/ml insulin, 0.5 μg/ml hydrocortisone (Sigma, St. Louis, MO), 100 ng/ml cholera toxin (Cambrex, Westborough, MA), and 100 units/ml penicillin and 100 μg/ml streptomycin (Invitrogen, Carlsbad, CA). A Growth Factor-Reduced MCF-10A Medium for the second step of transformation and culturing transformed clones was composed of DMEM/F12 supplemented with 5% donor horse serum (Sigma, St. Louis, MO) and 100 units/ml penicillin and 100 μg/ml streptomycin (Invitrogen, Carlsbad, CA), without the addition of supplements.

The acridine mutagen ICR191 (Sigma Chemical Co., St. Louis, MO) was dissolved at 1 mg/mL in 0.01 *M *HCl and was used as a stock solution before diluting in the tissue-culture medium at 500 ng/ml concentration.

### Transformation of MCF-10A cells using random mutagenesis and selection

Two steps of mutagenesis and selection had been used to achieve the transformation of MCF-10A cells. The first step of selection was for the ability to replicate despite DNA damage-response activation. In this step, 1 million MCF-10A cells were seeded in a 100 mm tissue-culture plate and cultivated for six months in Complete MCF-10A Growth Medium containing 500 ng/ml of ICR191. During this process, as cells reached confluence, they were trypsinized, and half the cells were frozen in liquid nitrogen. The remaining cells were returned to the culture. In the second step, cells were selected either for the ability to grow in the medium with reduced growth-factor supply, for the ability to grow in the hypoxic condition, or for anchorage-independent growth. To select cells resistant to a reduced growth-factor supply, cells were cultivated in the Growth Factor-Reduced MCF-10A Medium containing 500 ng/ml of ICR191 for one to three months. To select cells resistant to hypoxia, cells were cultivated in the Complete MCF-10A Growth Medium containing 100 μM of cobalt chloride, which is known to generate hypoxic environment [22], and 500 ng/ml of ICR191 for one month. To select for the ability for anchorage-independent growth, cells were challenged to grow in soft agar.

### Soft-agar cloning

1 × 10^5 ^cells in Complete MCF-10A Growth Medium were seeded into six-well plates with 2 ml 0.33% (w/v) low-melting agarose, which was overlaid onto 2 ml of 0.6% (w/v) agarose and fed every other day by exchanging 1.5 ml medium on top of the soft agar. Soft-agar cultures were maintained at 37°C and observed microscopically for the appearance of colonies. After three to four weeks in culture, the colonies were counted. In cases where cultures were to be established from anchorage-independent colonies, the top layer of low-melting agarose was plated into tissue-culture dishes and kept in Complete MCF-10A Growth Medium. Clones were picked using cloning cylinders and developed into cell-lines.

### Nude mice tumorigenicity assay

Cells in phosphate-buffered saline were mixed 1:1 with Matrigel basement membrane matrix (BD Biosciences, Franklin Lakes, NJ) and 3–4 × 10^6 ^cells in 100–150 μl were injected subcutaneously into the flanks of 5-week-old athymic female Ncr -nu/nu mice (National Cancer Institute). The animals were maintained in accordance with institutional guidelines at the Laboratory Animal Facility at RPCI for six to eight months and palpated weekly for tumor appearance. When tumor nodules reached 1–2 cm in size, the mice were euthanized by exposure to carbon dioxide. The tumors were excised and divided into three pieces for developing into cell-lines to be frozen for future microarray analysis, fixed in neutral buffered formalin and embedded in paraffin for a routine histological evaluation with 5 μm hematoxylin and eosin (H&E) stained sections. Histological procedures were performed at the RPCI Pathology Core Facility.

### GINI analysis

Identifying mutant genes in the transformed MCF-10A cells using inhibition of nonsense-mediated decay and Affymetrix oligonucletide U133Plus2.0 GeneChip analysis was performed as described previously for prostate cells [16]. Briefly, control untransformed MCF-10A cells and the MCF-10A transformed derivative cells were grown in Complete MCF-10A Growth Medium until they reached 70–80% of confluency. To inhibit NMD in cells, emetine (100 μg/ml) was added to the tissue-culture medium and, eight hours later, the total RNA was prepared with TRIZOL reagents (Invitrogen, Carlsbad, CA) according to manufacturer's specifications from emetine-treated as well as untreated cells. The integrity and purity of the total RNA were analyzed using a microfluidic electrophoretic system (Agilent 2100 Bioanalyzer; Agilent Technologies). RNA samples were processed and then hybridized to Human Genome U133APlus2.0 GeneChip (Affymetrix Inc, Santa Clara, CA), according to the manufacturer's instructions, by the Gene Expression Core facility at Roswell Park Cancer Institute. GeneChip expression array analysis was performed using Affymetrix Microarray Suite software, version 5 (GeneChip Analysis Suite). This software uses two independent sets of algorithms, a quantitative algorithm (robust estimator of the mean difference in probe intensities) that computes the raw signal and the SLR directly from the hybridization intensities of the probes, as well as a confidence algorithm (non-parametric) that provides P-values to estimate the confidence in detection (absent or present) or change (increase, decrease or no change) of a specific target.

To select candidate genes out of hundreds genes that show mRNA increase following emetine treatment in the transformed cells for sequencing analysis, we used the following cut-off criteria:1) the level of expression of a gene in the emetine-untreated transformed cells had to be more than threefold lower than that in emetine-untreated control MCF-10A cells; 2) the gene expression in control untreated MCF-10A cells had to be detected as present; 3) fold-change increase of mRNA level following emetine treatment in the transformed cells had to be at least threefold higher than that in the untransformed control cells.

### Sequencing analysis

One microgram of total RNA from emetine-treated transformed MCF-10A cells was reverse transcribed using the SuperScript II protocol (Invitrogen, Carlsbad, CA). Overlapping PCR primer sets were used to generate products spanning entire open-reading frames for candidate genes. Primers for sequencing analysis were designed using Primer3 software available online [23]. To sequence the p53 gene from genomic DNA, the sequences of p53 gene exons with the flanking introns were retrieved from the genomic databases, and the primers for PCR amplification of genomic DNA fragments of p53 gene were designed using Primer3 software. These are available upon request. The PCR products were gel-purified and sequenced using Applied Biosystems' PRISM 3100 Genetic Analyzer at the DNA Sequencing section of the Biopolymer Facility at RPCI.

### TP53 western blot analysis

Whole-cell extracts were prepared in a RIPA buffer, and protein samples were denatured, separated by SDS/PAGE gel, and transferred onto a Hybond-P nitrocellulose membrane (Amersham Pharmacia) by using the MiniPROTEAN 3 apparatus (Bio-Rad). Membranes were rinsed and blocked with 0.05% Tween 20 (Sigma) and 1% skimmed milk in PBS for one hour with three changes of this blocking buffer every 20 minutes at room temperature. Membranes were then incubated with DO1 antibody conjugated with horseredish peroxidase (Santa Cruz Biotechnology, Santa Cruz) in a blocking buffer. After washing the membranes in blocking buffer for one hour with a change of the buffer every 15 minutes at room temperature, membranes were visualized using ECL plus the Western blotting detection system (Amersham Pharmacia). The anti-actin monoclonal antibody (Sigma) was hybridized to membranes to serve as a measure of loading control.

### Flow cytometry analysis

After rinsing with a phosphate-buffered saline solution, the cells were trypsinized from culture dishes, rinsed with PBS, fixed in 70% ice-cold ethanol, and stained with 10 μg/ml propidium iodide (PI) for 30 minutes. Flow cytometry analysis was performed on the Coulter FACS analyzer equipped with an air-cooled argon laser, producing 488 nm light in which fluorescence light-emission is collected with a 620BP filter. An extended analysis of the DNA content and calculations of the percentage of cells in each phase of the cell-cycle were performed on ModFit Lt software (Verity Software House, Topsham, ME).

### SKY Analysis

Cell cultures usually received treatments with Colcemid (0.03–0.06 μg/ml). However, nocodazole (0.5 μg/ml) was found to give the best results with some cells. Chromosome spreads were prepared using air-drying methods. After sequential digestion with RNase and pepsin according to the procedure recommended by Applied Spectral Imaging, Inc. (ASI: Vista, CA 92081), the chromosomal DNA on the slides was denatured in 70% formamide and then hybridized with a cocktail of human SKY paint probes tagged with various nucleotide analogues (i.e., a mixture of individual chromosome DNA prepared by flow-sorting and PCR amplification)[24]. Thirty to 50 mitoses were chosen at random, and the images developed by combinations of five different flouorophores, such as Rhodamine, Texas-Red, Cy5, FITC and Cy5.5, were captured with a Spectral cube and Interferometer module installed on a Nikon microscope. Spectra-Karyotypes were carried out using SKY View software (Version 1.62).

### Array comparative genomic hybridization analysis

The preparation of Roswell Park Cancer Institute (RPCI) custom CGH arrays using RPCI-11 BACs has been described previously[25,26]. Briefly, the RPCI array contains ~6,000 RPCI-11 BAC clones that provide an average resolution across the genome of 420 kilobases. BACs were printed in duplicate on amino-silanated glass slides (Schott Nexterion, type A) using a MicroGrid ll TAS arrayer (Apogent Discoveries, Hudson, NH) to generate an array of ~12,000 elements. Genomic control MCF-10A DNA and the transformed MCF10AαL DNA were fluorescently labeled by random priming and hybridized, as described [25]. The hybridized slides were scanned using a Genepix 4200A scanner (Axon, Inc., Union City, CA) to generate high-resolution (10 μm) images for both Cy3 and Cy5 channels, and image analysis was performed using ImaGene (version 4.1) software (BioDiscovery, Inc., El Segundo, CA).

## Competing interests

The authors declare that they have no competing interests.

## Authors' contributions

HZ, DK and YI carried out all the experiments, LH performed Affymetrix microarray analysis, MV performed sequencing analysis, RTC performed immunohistochemistry and pathology analysis, SIM performed SKY nalaysis and YI conceived the study, participated in its design and coordination and drafted the manuscript. All authors read and approved the final manuscript
